# Letter from the Editor in Chief

**DOI:** 10.19102/icrm.2019.100705

**Published:** 2019-07-15

**Authors:** Moussa Mansour


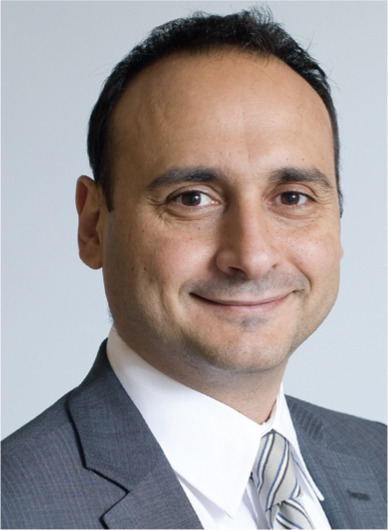


Dear Readers,

A unique feature of *The Journal of Innovations in Cardiac Rhythm Management* is its focus on the practical aspects of clinical cardiac electrophysiology. On a few occasions, however, preclinical articles are also published when they relate to an area of significant clinical interest. This issue contains one such article titled “Use of Light Sensor and Focused Local Atrial Electrogram Recordings for the Monitoring of Thermal Injury to the Esophagus and Lungs During Laser Catheter Ablation of the Posterior Atrial Walls: Preclinical In Vitro Porcine and In Vivo Canine Experimental Studies.”^1^ In it, the authors describe the use of a novel technique for the protection of the esophagus during ablation for atrial fibrillation (AF) evaluated in a series of animal experiments.

While this article describes a technology in its early form, it remains important because it highlights a central issue in AF ablation, which is esophageal injury. This complication is the most devastating adverse event associated with AF ablation and has been found to occur with the application of all commercially available energy sources currently used for ablation. Methods that are used at this time to reduce the risk of injury to the esophagus include temperature measurement and mechanical displacement of the esophagus. However clinical data regarding the efficacy of these approaches are lacking. More importantly, defining “safe” parameters for ablation continues to be an elusive target.

The task of proving the efficacy of esophageal protection tools is not easy. The incidence of injury is low, which means that studies enrolling thousands of patients are needed in order to show an effect. As a result, randomized studies are difficult to conduct. However, despite the existing difficulty, performing such studies is critical. Perhaps softer and more common endpoints such as endoscopy findings can be used to facilitate the completion of these studies. Performing clinical studies aimed at eliminating this complication is critical not only because of its severity but also because its incidence will likely increase in the future with the development of ablation tools designed to establish more durable pulmonary vein isolation lesions.

Sincerely,


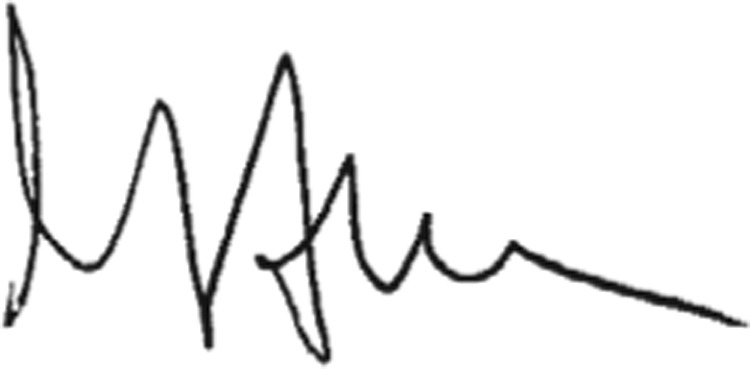


Moussa Mansour, md, fhrs, facc

Editor in Chief

The Journal of Innovations in Cardiac Rhythm Management

MMansour@InnovationsInCRM.com

Director, Atrial Fibrillation Program

Jeremy Ruskin and Dan Starks Endowed Chair in Cardiology

Massachusetts General Hospital

Boston, MA 02114
